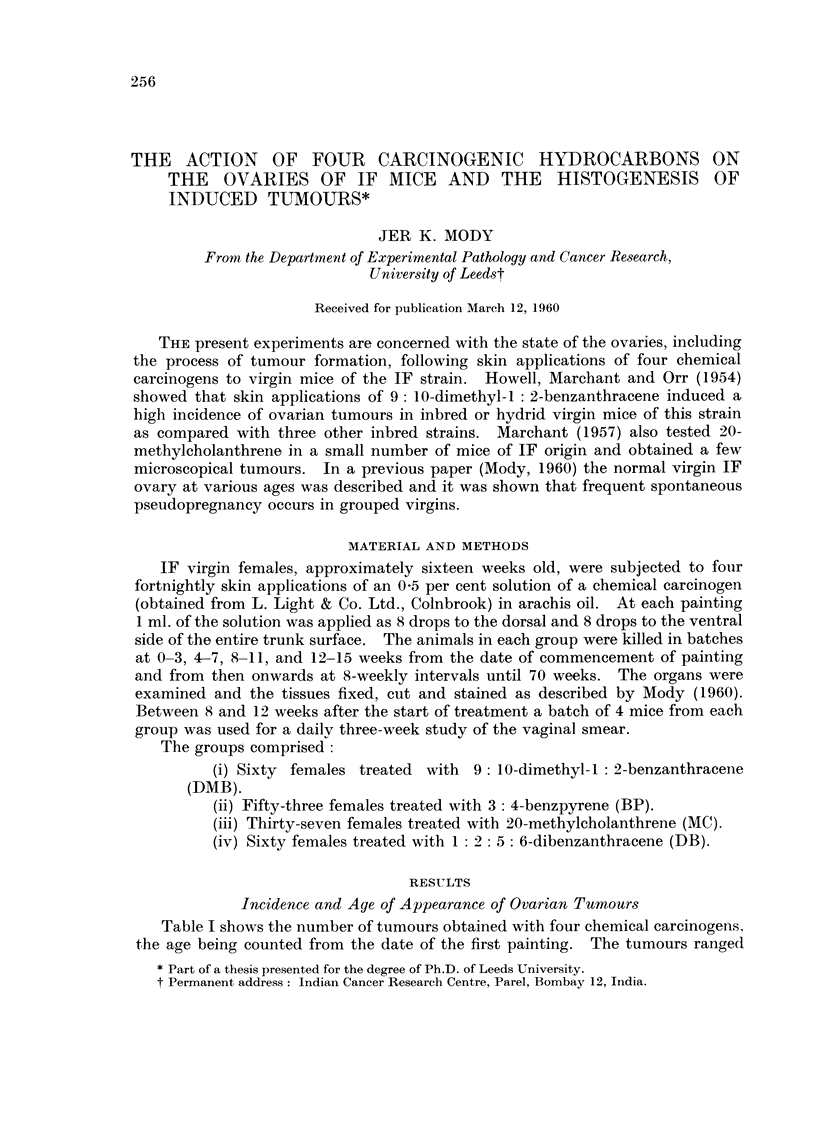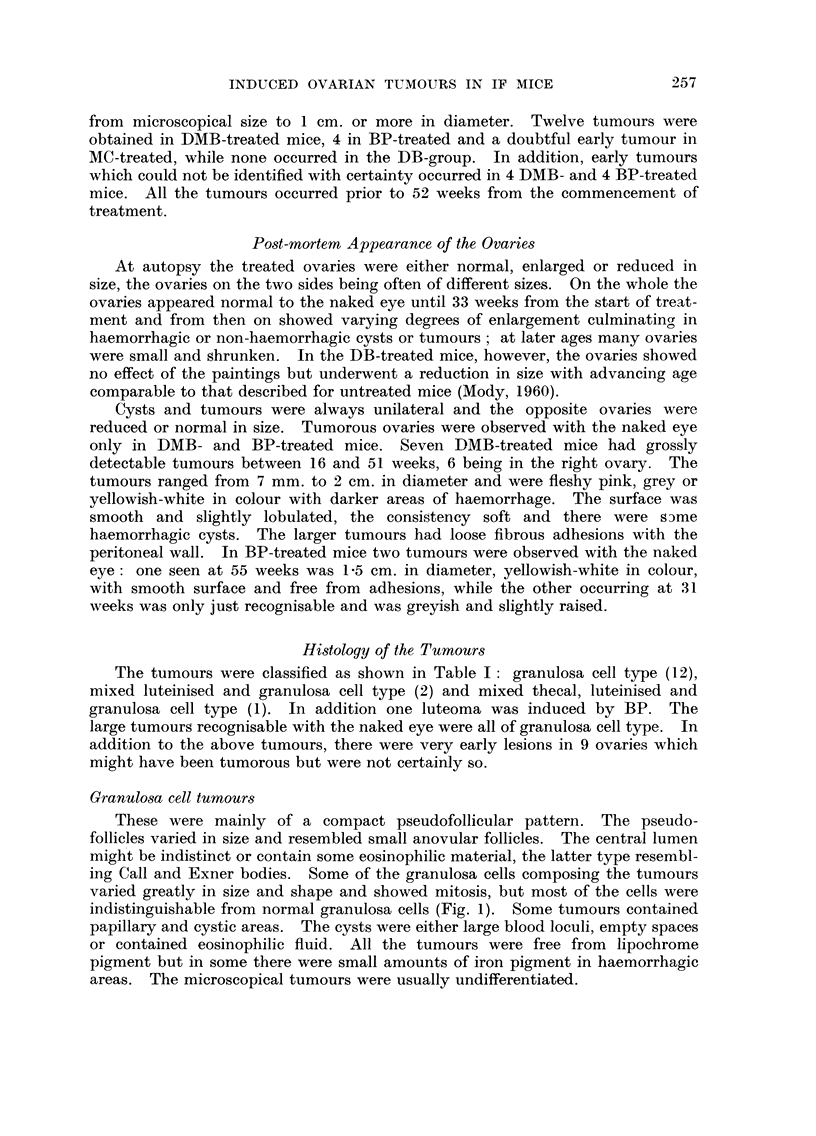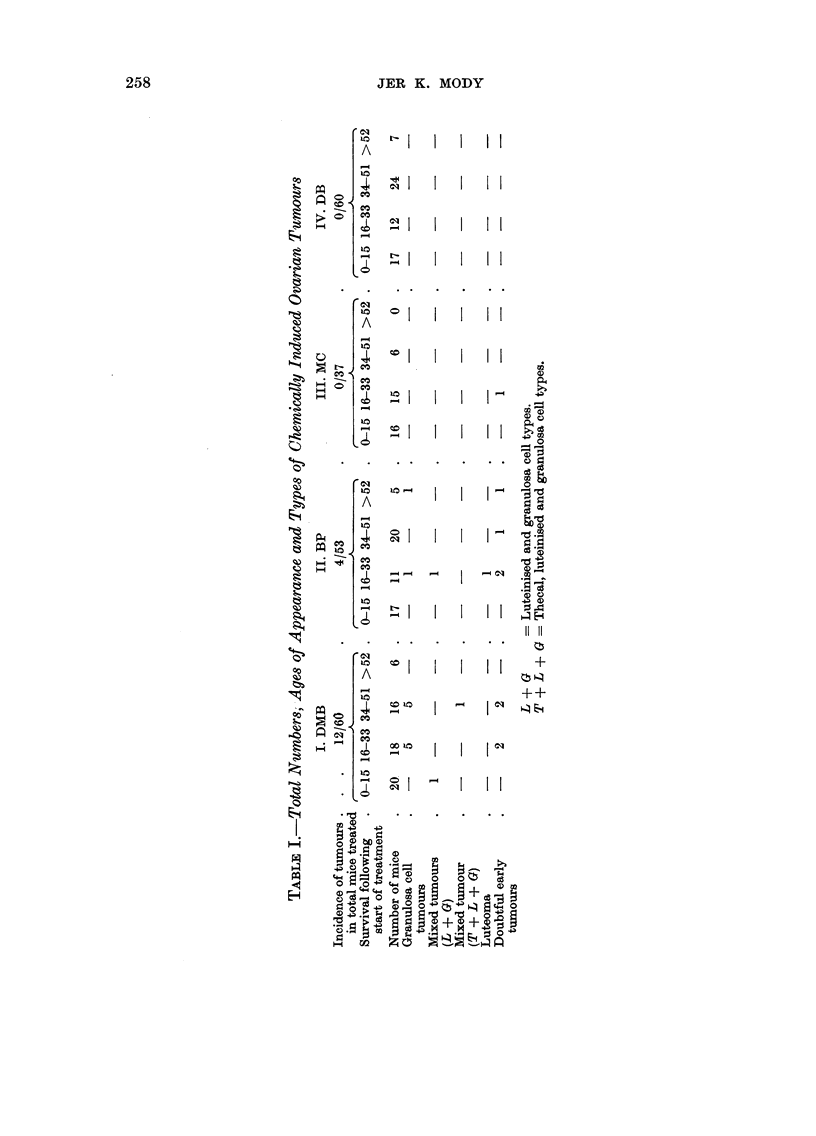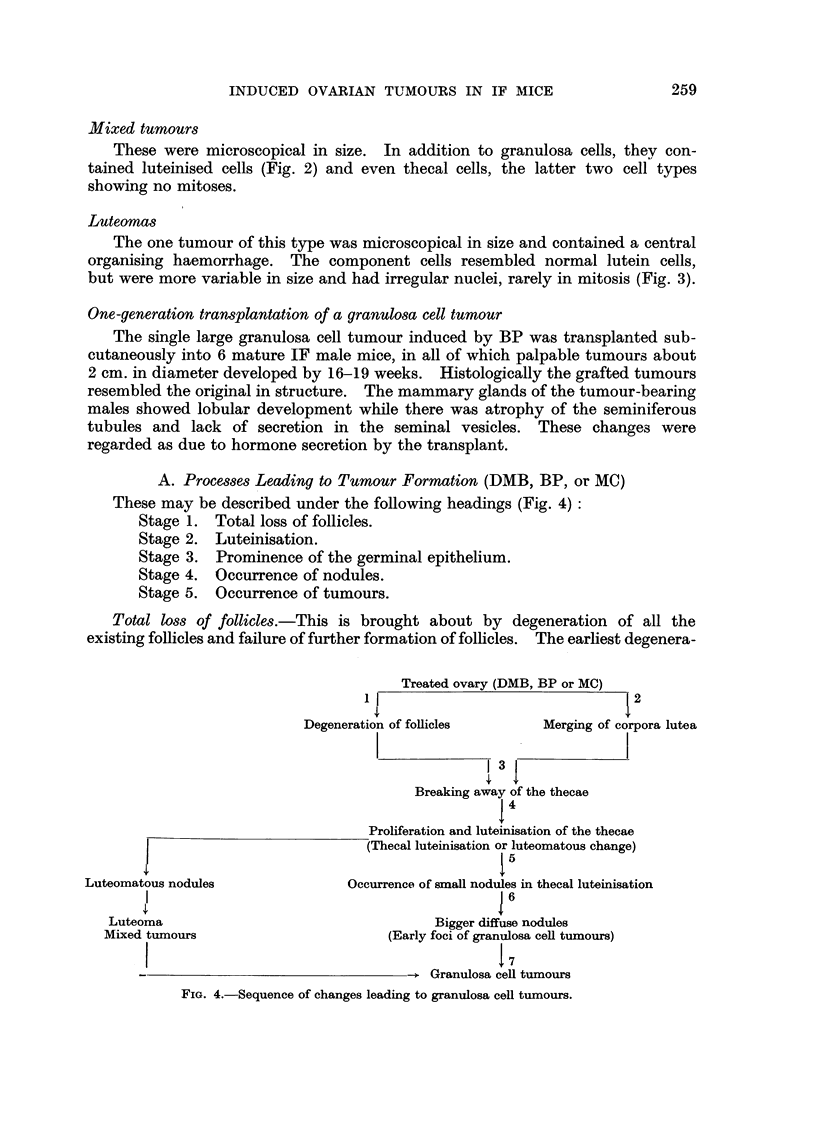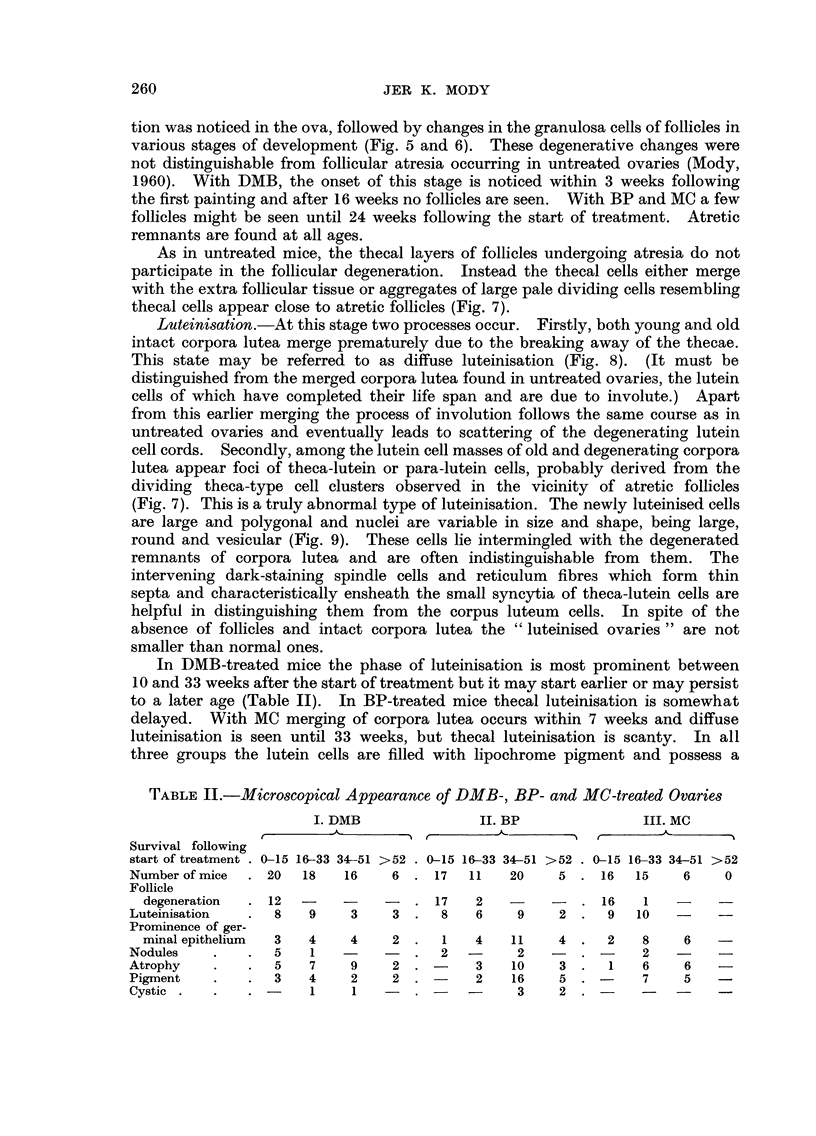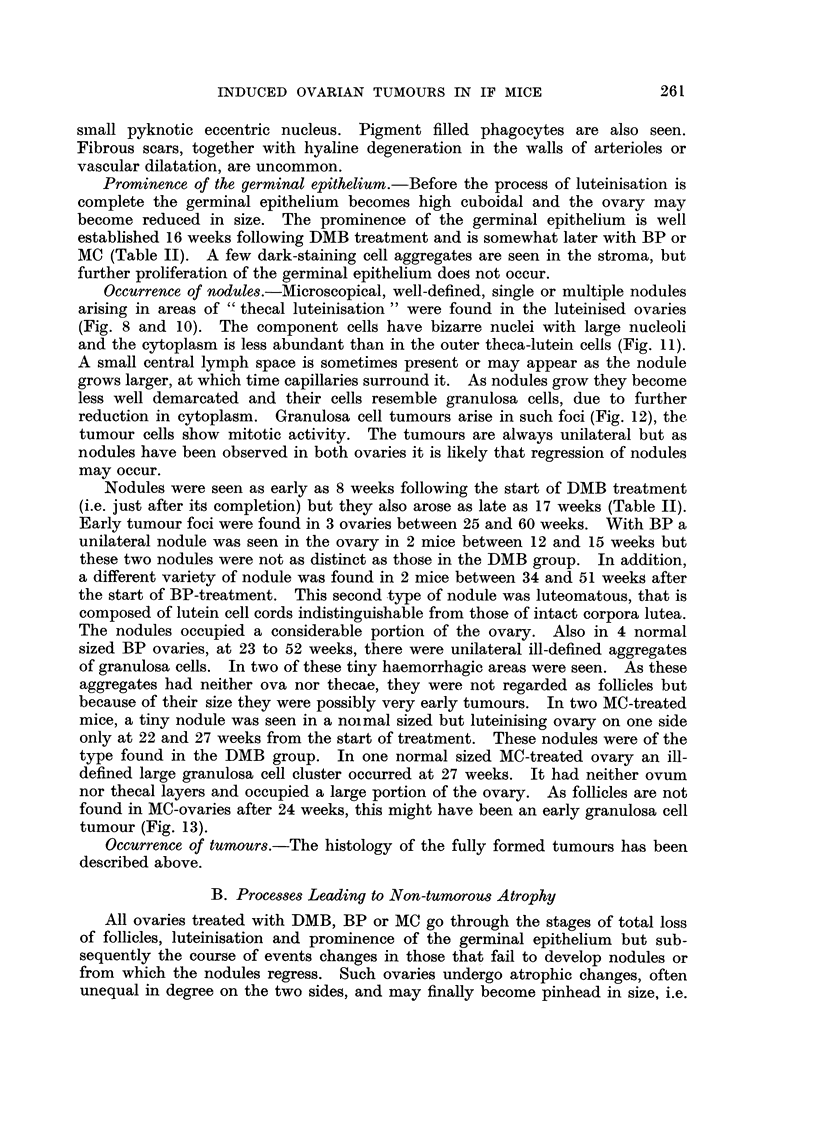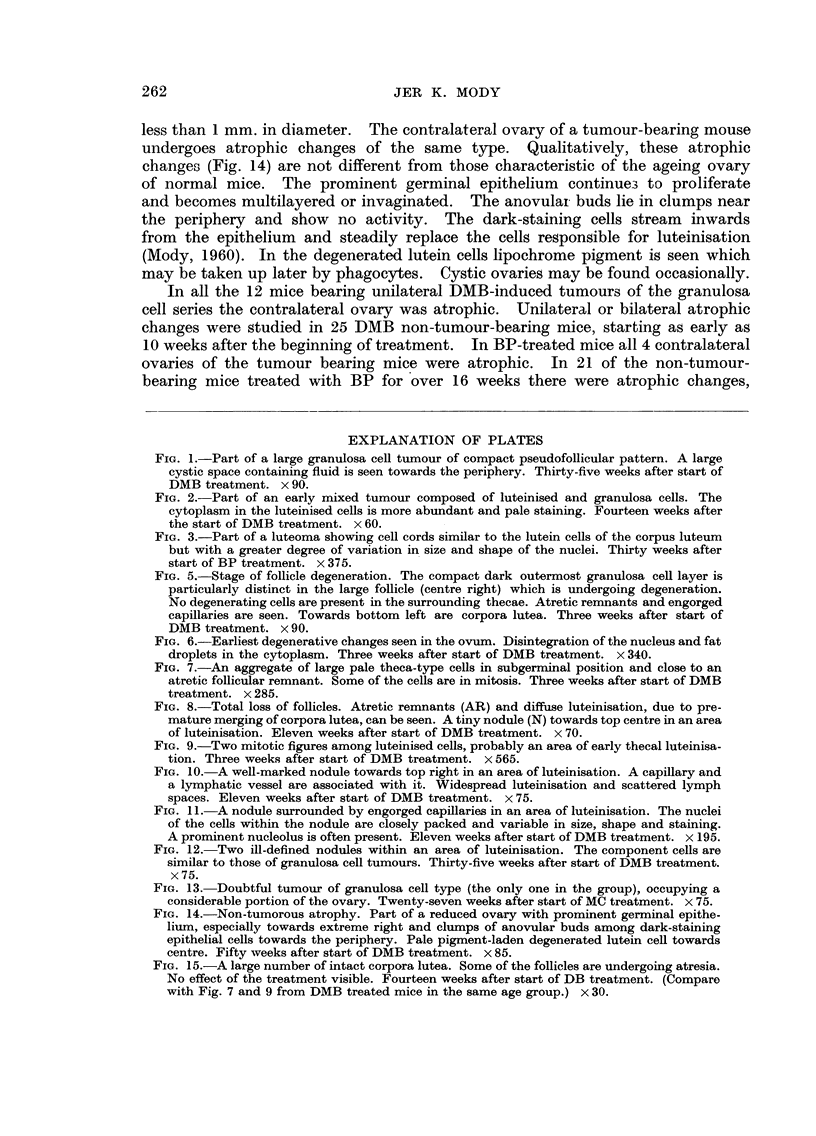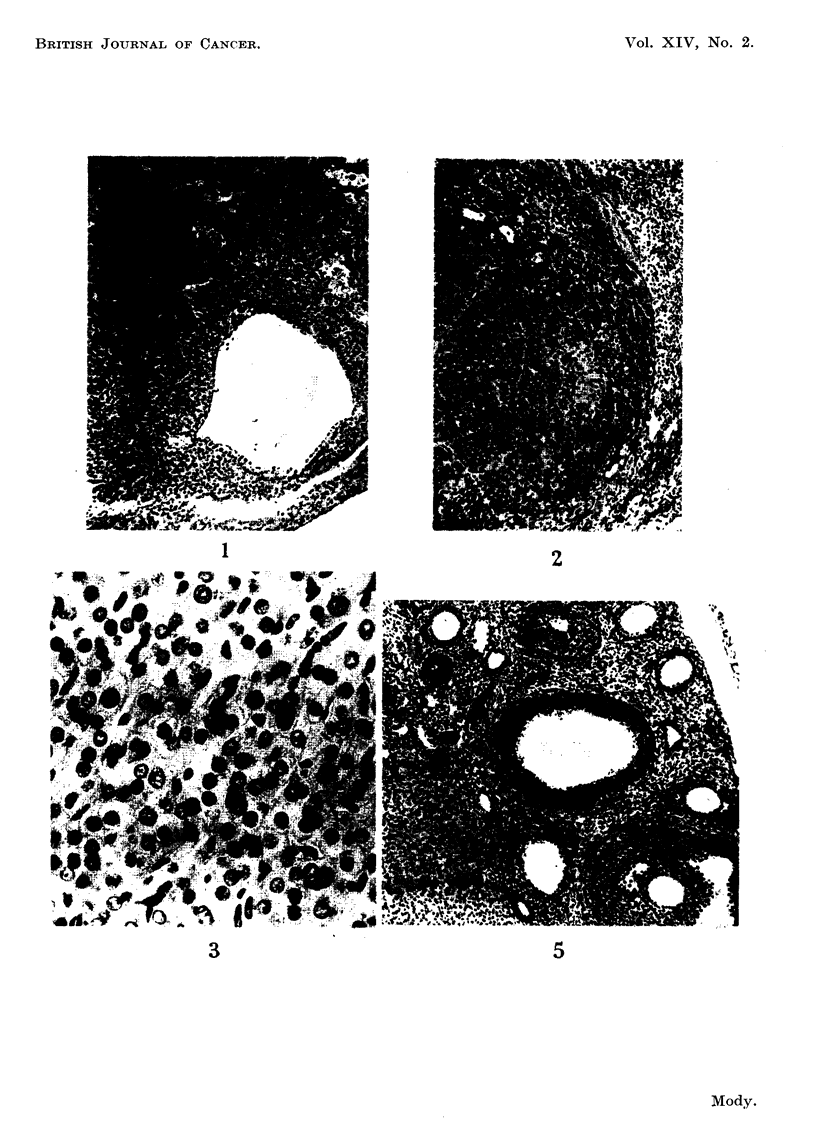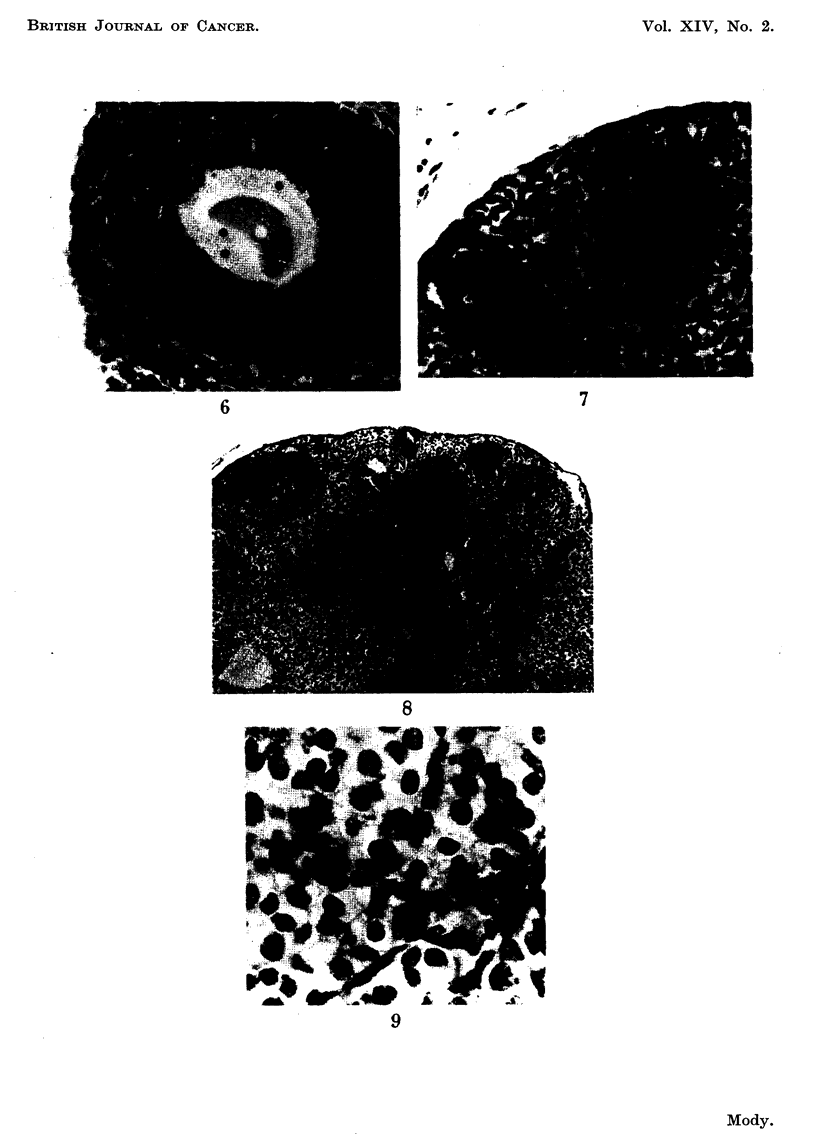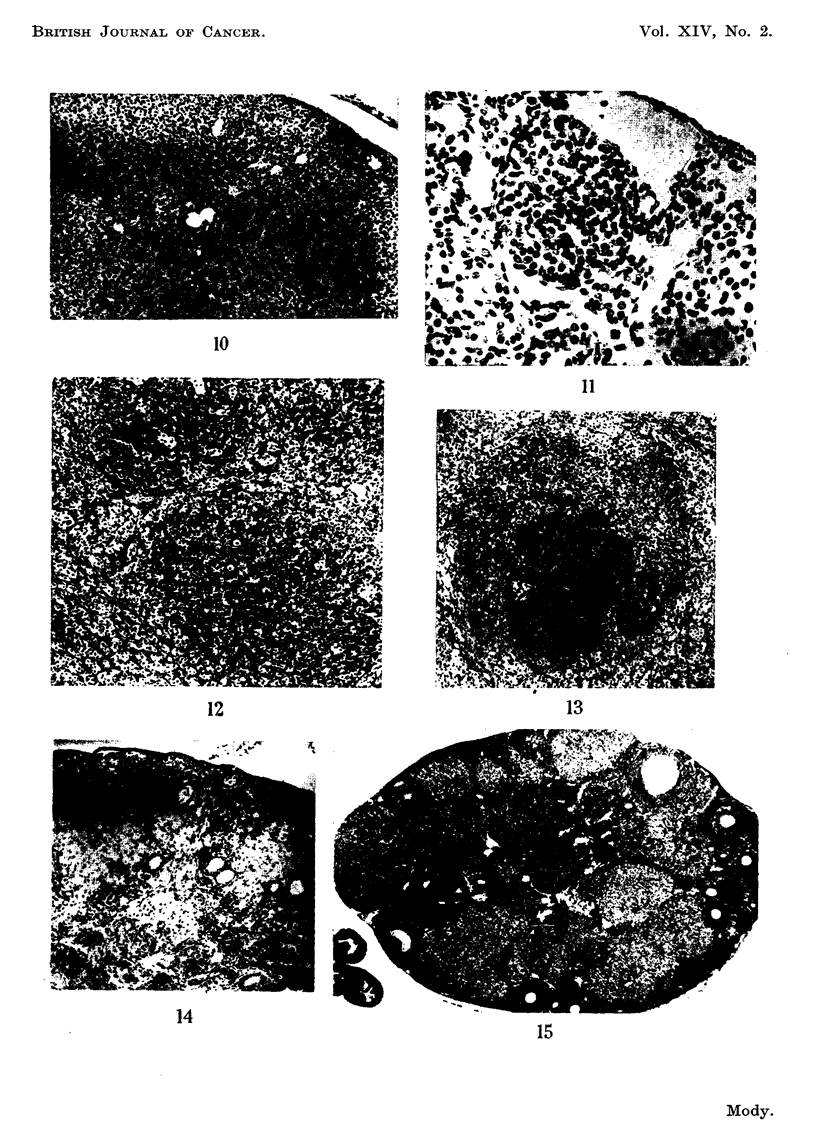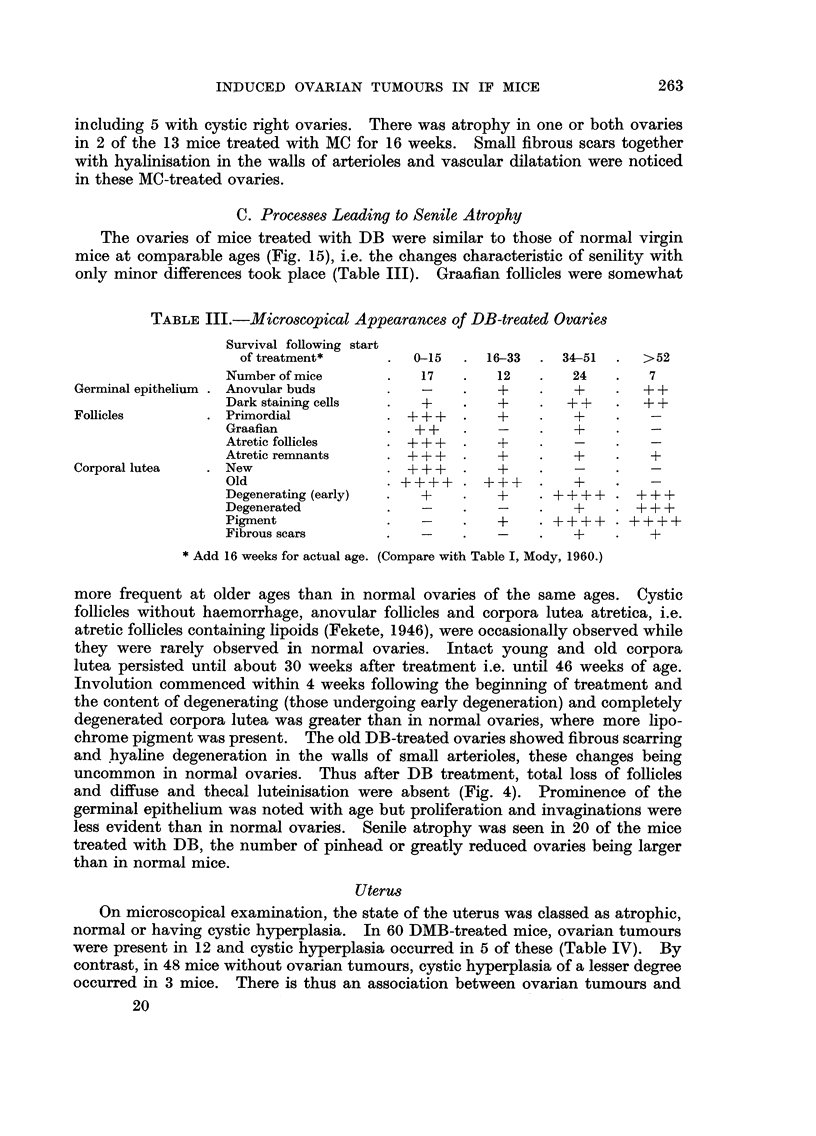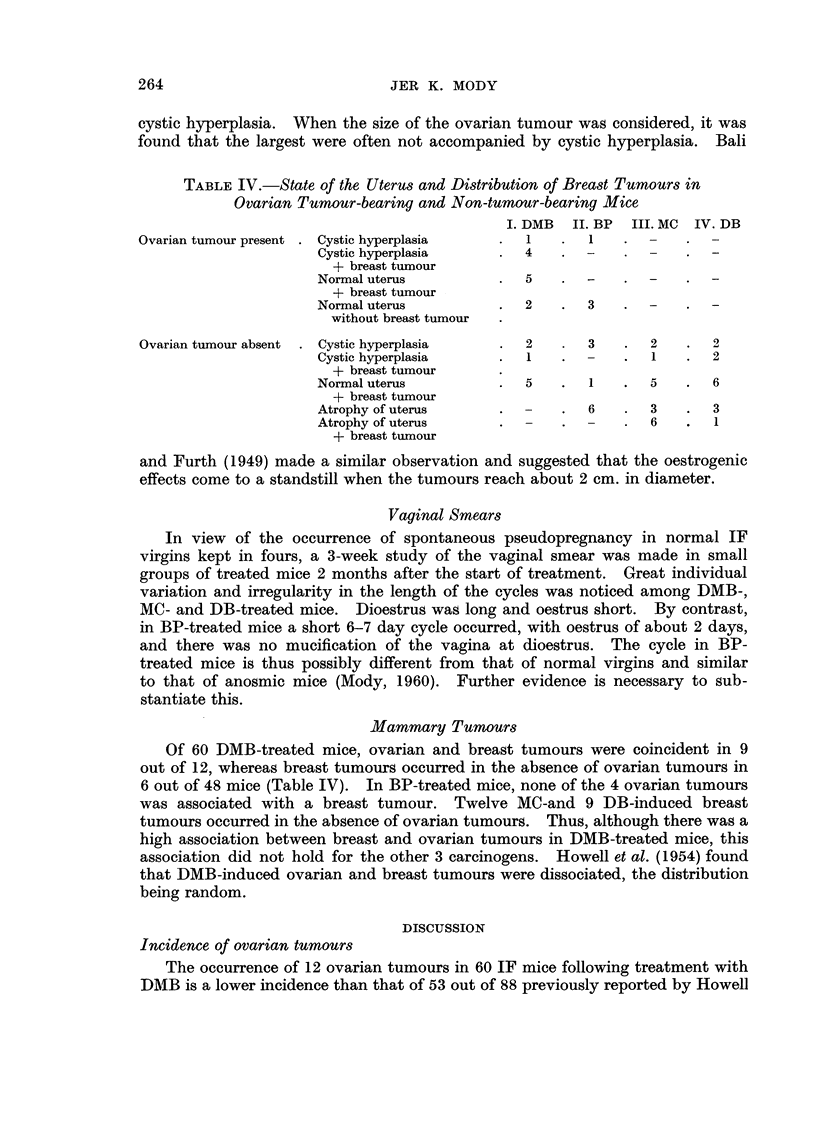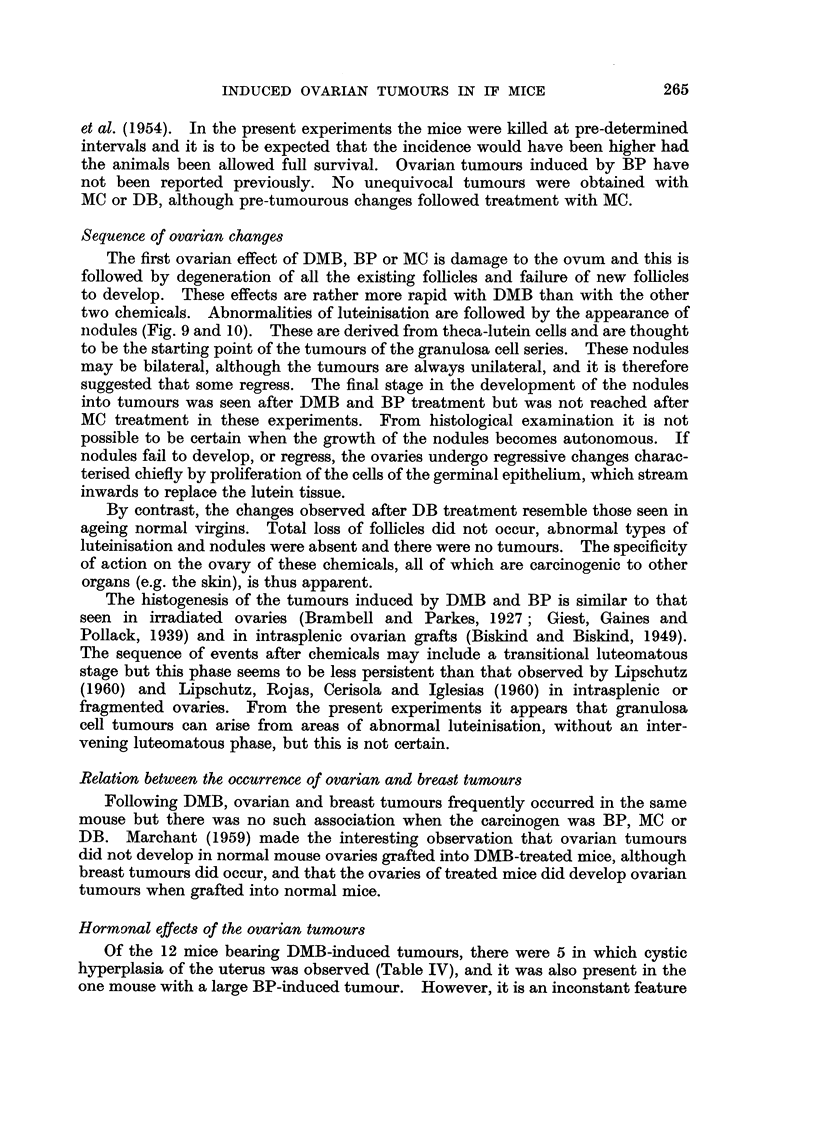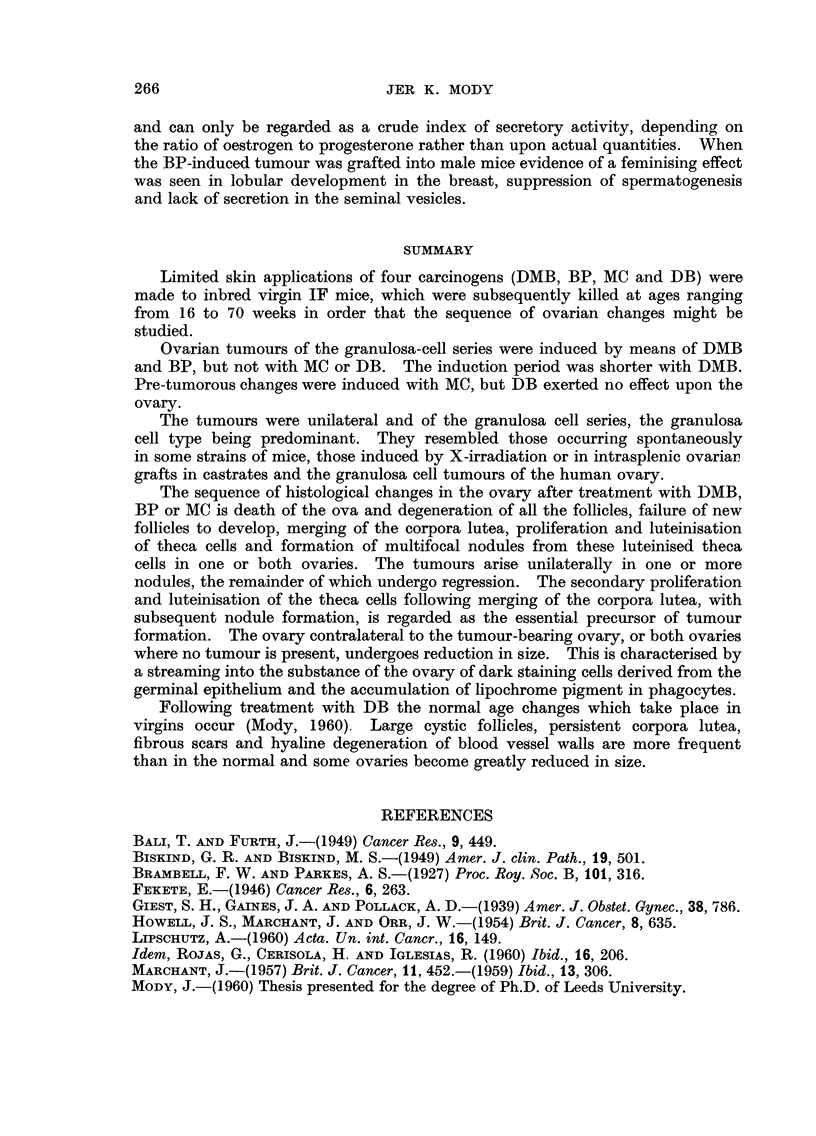# The Action of Four Carcinogenic Hydrocarbons on the Ovaries of IF Mice and the Histogenesis of Induced Tumours*

**DOI:** 10.1038/bjc.1960.30

**Published:** 1960-06

**Authors:** Jer K. Mody

## Abstract

**Images:**


### THE ACTION OF FOUR CARCINOGENIC HYDROCARBONS ON THE OVARIES OF IF MICE AND THE HISTOGENESIS OF INDUCED TUMOURS*

JER K. MODY

*From the Department of Experimental Pathology and Cancer Research, University of Leeds*†

Received for publication March 12, 1960

THE present experiments are concerned with the state of the ovaries, including the process of tumour formation, following skin applications of four chemical carcinogens to virgin mice of the IF strain. Howell, Marchant and Orr (1954) showed that skin applications of 9 : 10-dimethyl-1 : 2-benzanthracene induced a high incidence of ovarian tumours in inbred or hydrid virgin mice of this strain as compared with three other inbred strains. Marchant (1957) also tested 20-methylcholanthrene in a small number of mice of IF origin and obtained a few microscopical tumours. In a previous paper (Mody, 1960) the normal virgin IF ovary at various ages was described and it was shown that frequent spontaneous pseudopregnancy occurs in grouped virgins.

### MATERIAL AND METHODS

IF virgin females, approximately sixteen weeks old, were subjected to four fortnightly skin applications of an 0·5 per cent solution of a chemical carcinogen (obtained from L. Light & Co. Ltd., Colnbrook) in arachis oil. At each painting 1 ml. of the solution was applied as 8 drops to the dorsal and 8 drops to the ventral side of the entire trunk surface. The animals in each group were killed in batches at 0–3, 4–7, 8–11, and 12–15 weeks from the date of commencement of painting and from then onwards at 8-weekly intervals until 70 weeks. The organs were examined and the tissues fixed, cut and stained as described by Mody (1960). Between 8 and 12 weeks after the start of treatment a batch of 4 mice from each group was used for a daily three-week study of the vaginal smear.

The groups comprised :

(i) Sixty females treated with 9 : 10-dimethyl-1 : 2-benzanthracene (DMB).

(ii) Fifty-three females treated with 3 : 4-benzpyrene (BP).

(iii) Thirty-seven females treated with 20-methylcholanthrene (MC).

(iv) Sixty females treated with 1 : 2 : 5 : 6-dibenzanthracene (DB).

### RESULTS

### *Incidence and Age of Appearance of Ovarian Tumours*

Table I shows the number of tumours obtained with four chemical carcinogens, the age being counted from the date of the first painting. The tumours ranged

* Part of a thesis presented for the degree of Ph.D. of Leeds University.

† Permanent address : Indian Cancer Research Centre, Parel, Bombay 12, India.

from microscopical size to 1 cm. or more in diameter. Twelve tumours were obtained in DMB-treated mice, 4 in BP-treated and a doubtful early tumour in MC-treated, while none occurred in the DB-group. In addition, early tumours which could not be identified with certainty occurred in 4 DMB- and 4 BP-treated mice. All the tumours occurred prior to 52 weeks from the commencement of treatment.

### *Post-mortem Appearance of the Ovaries*

At autopsy the treated ovaries were either normal, enlarged or reduced in size, the ovaries on the two sides being often of different sizes. On the whole the ovaries appeared normal to the naked eye until 33 weeks from the start of treatment and from then on showed varying degrees of enlargement culminating in haemorrhagic or non-haemorrhagic cysts or tumours ; at later ages many ovaries were small and shrunken. In the DB-treated mice, however, the ovaries showed no effect of the paintings but underwent a reduction in size with advancing age comparable to that described for untreated mice (Mody, 1960).

Cysts and tumours were always unilateral and the opposite ovaries were reduced or normal in size. Tumorous ovaries were observed with the naked eye only in DMB- and BP-treated mice. Seven DMB-treated mice had grossly detectable tumours between 16 and 51 weeks, 6 being in the right ovary. The tumours ranged from 7 mm. to 2 cm. in diameter and were fleshy pink, grey or yellowish-white in colour with darker areas of haemorrhage. The surface was smooth and slightly lobulated, the consistency soft and there were some haemorrhagic cysts. The larger tumours had loose fibrous adhesions with the peritoneal wall. In BP-treated mice two tumours were observed with the naked eye : one seen at 55 weeks was 1·5 cm. in diameter, yellowish-white in colour, with smooth surface and free from adhesions, while the other occurring at 31 weeks was only just recognisable and was greyish and slightly raised.

### *Histology of the Tumours*

The tumours were classified as shown in Table I : granulosa cell type (12), mixed luteinised and granulosa cell type (2) and mixed thecal, luteinised and granulosa cell type (1). In addition one luteoma was induced by BP. The large tumours recognisable with the naked eye were all of granulosa cell type. In addition to the above tumours, there were very early lesions in 9 ovaries which might have been tumorous but were not certainly so.

#### *Granulosa cell tumours*

These were mainly of a compact pseudofollicular pattern. The pseudofollicles varied in size and resembled small anovular follicles. The central lumen might be indistinct or contain some eosinophilic material, the latter type resembling Call and Exner bodies. Some of the granulosa cells composing the tumours varied greatly in size and shape and showed mitosis, but most of the cells were indistinguishable from normal granulosa cells (Fig. 1). Some tumours contained papillary and cystic areas. The cysts were either large blood loculi, empty spaces or contained eosinophilic fluid. All the tumours were free from lipochrome pigment but in some there were small amounts of iron pigment in haemorrhagic areas. The microscopical tumours were usually undifferentiated.

TABLE I.—*Total Numbers, Ages of Appearance and Types of Chemically Induced Ovarian Tumours*

| | I. DMB | | | | II. BP | | | | III. MC | | | | IV. DB | | | |
|---|---|---|---|---|---|---|---|---|---|---|---|---|---|---|---|---|
| Incidence of tumours in total mice treated | 12/60 | | | | 4/53 | | | | 0/37 | | | | 0/60 | | | |
| Survival following start of treatment | 0–15 | 16–33 | 34–51 | >52 | 0–15 | 16–33 | 34–51 | >52 | 0–15 | 16–33 | 34–51 | >52 | 0–15 | 16–33 | 34–51 | >52 |
| Number of mice | 20 | 18 | 16 | 6 | 17 | 11 | 20 | 5 | 16 | 15 | 6 | 0 | 17 | 12 | 24 | 7 |
| Granulosa cell tumours | — | 5 | 5 | — | — | 1 | — | 1 | — | — | — | — | — | — | — | — |
| Mixed tumours ($L + G$) | 1 | — | — | — | — | 1 | — | — | — | — | — | — | — | — | — | — |
| Mixed tumour ($T + L + G$) | — | — | 1 | — | — | — | — | — | — | — | — | — | — | — | — | — |
| Luteoma | — | — | — | — | — | 1 | — | — | — | — | — | — | — | — | — | — |
| Doubtful early tumours | — | 2 | 2 | — | — | 2 | 1 | 1 | — | 1 | — | — | — | — | — | — |

$L + G$ = Luteinised and granulosa cell types.
$T + L + G$ = Thecal, luteinised and granulosa cell types.

*Mixed tumours*

These were microscopical in size. In addition to granulosa cells, they contained luteinised cells (Fig. 2) and even thecal cells, the latter two cell types showing no mitoses.

*Luteomas*

The one tumour of this type was microscopical in size and contained a central organising haemorrhage. The component cells resembled normal lutein cells, but were more variable in size and had irregular nuclei, rarely in mitosis (Fig. 3).

*One-generation transplantation of a granulosa cell tumour*

The single large granulosa cell tumour induced by BP was transplanted subcutaneously into 6 mature IF male mice, in all of which palpable tumours about 2 cm. in diameter developed by 16–19 weeks. Histologically the grafted tumours resembled the original in structure. The mammary glands of the tumour-bearing males showed lobular development while there was atrophy of the seminiferous tubules and lack of secretion in the seminal vesicles. These changes were regarded as due to hormone secretion by the transplant.

A. *Processes Leading to Tumour Formation* (DMB, BP, or MC)

These may be described under the following headings (Fig. 4) :

Stage 1. Total loss of follicles.
Stage 2. Luteinisation.
Stage 3. Prominence of the germinal epithelium.
Stage 4. Occurrence of nodules.
Stage 5. Occurrence of tumours.

*Total loss of follicles.*—This is brought about by degeneration of all the existing follicles and failure of further formation of follicles. The earliest degenera-

Fig. 4.—Sequence of changes leading to granulosa cell tumours.

tion was noticed in the ova, followed by changes in the granulosa cells of follicles in various stages of development (Fig. 5 and 6). These degenerative changes were not distinguishable from follicular atresia occurring in untreated ovaries (Mody, 1960). With DMB, the onset of this stage is noticed within 3 weeks following the first painting and after 16 weeks no follicles are seen. With BP and MC a few follicles might be seen until 24 weeks following the start of treatment. Atretic remnants are found at all ages.

As in untreated mice, the thecal layers of follicles undergoing atresia do not participate in the follicular degeneration. Instead the thecal cells either merge with the extra follicular tissue or aggregates of large pale dividing cells resembling thecal cells appear close to atretic follicles (Fig. 7).

*Luteinisation.*—At this stage two processes occur. Firstly, both young and old intact corpora lutea merge prematurely due to the breaking away of the thecae. This state may be referred to as diffuse luteinisation (Fig. 8). (It must be distinguished from the merged corpora lutea found in untreated ovaries, the lutein cells of which have completed their life span and are due to involute.) Apart from this earlier merging the process of involution follows the same course as in untreated ovaries and eventually leads to scattering of the degenerating lutein cell cords. Secondly, among the lutein cell masses of old and degenerating corpora lutea appear foci of theca-lutein or para-lutein cells, probably derived from the dividing theca-type cell clusters observed in the vicinity of atretic follicles (Fig. 7). This is a truly abnormal type of luteinisation. The newly luteinised cells are large and polygonal and nuclei are variable in size and shape, being large, round and vesicular (Fig. 9). These cells lie intermingled with the degenerated remnants of corpora lutea and are often indistinguishable from them. The intervening dark-staining spindle cells and reticulum fibres which form thin septa and characteristically ensheath the small syncytia of theca-lutein cells are helpful in distinguishing them from the corpus luteum cells. In spite of the absence of follicles and intact corpora lutea the " luteinised ovaries " are not smaller than normal ones.

In DMB-treated mice the phase of luteinisation is most prominent between 10 and 33 weeks after the start of treatment but it may start earlier or may persist to a later age (Table II). In BP-treated mice thecal luteinisation is somewhat delayed. With MC merging of corpora lutea occurs within 7 weeks and diffuse luteinisation is seen until 33 weeks, but thecal luteinisation is scanty. In all three groups the lutein cells are filled with lipochrome pigment and possess a

TABLE II.—*Microscopical Appearance of DMB-, BP- and MC-treated Ovaries*

| | I. DMB | | | | II. BP | | | | III. MC | | | |
|---|---|---|---|---|---|---|---|---|---|---|---|---|
| Survival following start of treatment | 0–15 | 16–33 | 34–51 | >52 | 0–15 | 16–33 | 34–51 | >52 | 0–15 | 16–33 | 34–51 | >52 |
| Number of mice | 20 | 18 | 16 | 6 | 17 | 11 | 20 | 5 | 16 | 15 | 6 | 0 |
| Follicle degeneration | 12 | — | — | — | 17 | 2 | — | — | 16 | 1 | — | — |
| Luteinisation | 8 | 9 | 3 | 3 | 8 | 6 | 9 | 2 | 9 | 10 | — | — |
| Prominence of germinal epithelium | 3 | 4 | 4 | 2 | 1 | 4 | 11 | 4 | 2 | 8 | 6 | — |
| Nodules | 5 | 1 | — | — | 2 | — | 2 | — | — | 2 | — | — |
| Atrophy | 5 | 7 | 9 | 2 | — | 3 | 10 | 3 | 1 | 6 | 6 | — |
| Pigment | 3 | 4 | 2 | 2 | — | 2 | 16 | 5 | — | 7 | 5 | — |
| Cystic | — | 1 | 1 | — | — | — | 3 | 2 | — | — | — | — |

small pyknotic eccentric nucleus. Pigment filled phagocytes are also seen. Fibrous scars, together with hyaline degeneration in the walls of arterioles or vascular dilatation, are uncommon.

*Prominence of the germinal epithelium.*—Before the process of luteinisation is complete the germinal epithelium becomes high cuboidal and the ovary may become reduced in size. The prominence of the germinal epithelium is well established 16 weeks following DMB treatment and is somewhat later with BP or MC (Table II). A few dark-staining cell aggregates are seen in the stroma, but further proliferation of the germinal epithelium does not occur.

*Occurrence of nodules.*—Microscopical, well-defined, single or multiple nodules arising in areas of " thecal luteinisation " were found in the luteinised ovaries (Fig. 8 and 10). The component cells have bizarre nuclei with large nucleoli and the cytoplasm is less abundant than in the outer theca-lutein cells (Fig. 11). A small central lymph space is sometimes present or may appear as the nodule grows larger, at which time capillaries surround it. As nodules grow they become less well demarcated and their cells resemble granulosa cells, due to further reduction in cytoplasm. Granulosa cell tumours arise in such foci (Fig. 12), the tumour cells show mitotic activity. The tumours are always unilateral but as nodules have been observed in both ovaries it is likely that regression of nodules may occur.

Nodules were seen as early as 8 weeks following the start of DMB treatment (i.e. just after its completion) but they also arose as late as 17 weeks (Table II). Early tumour foci were found in 3 ovaries between 25 and 60 weeks. With BP a unilateral nodule was seen in the ovary in 2 mice between 12 and 15 weeks but these two nodules were not as distinct as those in the DMB group. In addition, a different variety of nodule was found in 2 mice between 34 and 51 weeks after the start of BP-treatment. This second type of nodule was luteomatous, that is composed of lutein cell cords indistinguishable from those of intact corpora lutea. The nodules occupied a considerable portion of the ovary. Also in 4 normal sized BP ovaries, at 23 to 52 weeks, there were unilateral ill-defined aggregates of granulosa cells. In two of these tiny haemorrhagic areas were seen. As these aggregates had neither ova nor thecae, they were not regarded as follicles but because of their size they were possibly very early tumours. In two MC-treated mice, a tiny nodule was seen in a noımal sized but luteinising ovary on one side only at 22 and 27 weeks from the start of treatment. These nodules were of the type found in the DMB group. In one normal sized MC-treated ovary an ill-defined large granulosa cell cluster occurred at 27 weeks. It had neither ovum nor thecal layers and occupied a large portion of the ovary. As follicles are not found in MC-ovaries after 24 weeks, this might have been an early granulosa cell tumour (Fig. 13).

*Occurrence of tumours.*—The histology of the fully formed tumours has been described above.

### B. *Processes Leading to Non-tumorous Atrophy*

All ovaries treated with DMB, BP or MC go through the stages of total loss of follicles, luteinisation and prominence of the germinal epithelium but subsequently the course of events changes in those that fail to develop nodules or from which the nodules regress. Such ovaries undergo atrophic changes, often unequal in degree on the two sides, and may finally become pinhead in size, i.e.

less than 1 mm. in diameter. The contralateral ovary of a tumour-bearing mouse undergoes atrophic changes of the same type. Qualitatively, these atrophic changes (Fig. 14) are not different from those characteristic of the ageing ovary of normal mice. The prominent germinal epithelium continues to proliferate and becomes multilayered or invaginated. The anovular buds lie in clumps near the periphery and show no activity. The dark-staining cells stream inwards from the epithelium and steadily replace the cells responsible for luteinisation (Mody, 1960). In the degenerated lutein cells lipochrome pigment is seen which may be taken up later by phagocytes. Cystic ovaries may be found occasionally.

In all the 12 mice bearing unilateral DMB-induced tumours of the granulosa cell series the contralateral ovary was atrophic. Unilateral or bilateral atrophic changes were studied in 25 DMB non-tumour-bearing mice, starting as early as 10 weeks after the beginning of treatment. In BP-treated mice all 4 contralateral ovaries of the tumour bearing mice were atrophic. In 21 of the non-tumour-bearing mice treated with BP for over 16 weeks there were atrophic changes,

---

### EXPLANATION OF PLATES

FIG. 1.—Part of a large granulosa cell tumour of compact pseudofollicular pattern. A large cystic space containing fluid is seen towards the periphery. Thirty-five weeks after start of DMB treatment. ×90.

FIG. 2.—Part of an early mixed tumour composed of luteinised and granulosa cells. The cytoplasm in the luteinised cells is more abundant and pale staining. Fourteen weeks after the start of DMB treatment. ×60.

FIG. 3.—Part of a luteoma showing cell cords similar to the lutein cells of the corpus luteum but with a greater degree of variation in size and shape of the nuclei. Thirty weeks after start of BP treatment. ×375.

FIG. 5.—Stage of follicle degeneration. The compact dark outermost granulosa cell layer is particularly distinct in the large follicle (centre right) which is undergoing degeneration. No degenerating cells are present in the surrounding thecae. Atretic remnants and engorged capillaries are seen. Towards bottom left are corpora lutea. Three weeks after start of DMB treatment. ×90.

FIG. 6.—Earliest degenerative changes seen in the ovum. Disintegration of the nucleus and fat droplets in the cytoplasm. Three weeks after start of DMB treatment. ×340.

FIG. 7.—An aggregate of large pale theca-type cells in subgerminal position and close to an atretic follicular remnant. Some of the cells are in mitosis. Three weeks after start of DMB treatment. ×285.

FIG. 8.—Total loss of follicles. Atretic remnants (AR) and diffuse luteinisation, due to premature merging of corpora lutea, can be seen. A tiny nodule (N) towards top centre in an area of luteinisation. Eleven weeks after start of DMB treatment. ×70.

FIG. 9.—Two mitotic figures among luteinised cells, probably an area of early thecal luteinisation. Three weeks after start of DMB treatment. ×565.

FIG. 10.—A well-marked nodule towards top right in an area of luteinisation. A capillary and a lymphatic vessel are associated with it. Widespread luteinisation and scattered lymph spaces. Eleven weeks after start of DMB treatment. ×75.

FIG. 11.—A nodule surrounded by engorged capillaries in an area of luteinisation. The nuclei of the cells within the nodule are closely packed and variable in size, shape and staining. A prominent nucleolus is often present. Eleven weeks after start of DMB treatment. ×195.

FIG. 12.—Two ill-defined nodules within an area of luteinisation. The component cells are similar to those of granulosa cell tumours. Thirty-five weeks after start of DMB treatment. ×75.

FIG. 13.—Doubtful tumour of granulosa cell type (the only one in the group), occupying a considerable portion of the ovary. Twenty-seven weeks after start of MC treatment. ×75.

FIG. 14.—Non-tumorous atrophy. Part of a reduced ovary with prominent germinal epithelium, especially towards extreme right and clumps of anovular buds among dark-staining epithelial cells towards the periphery. Pale pigment-laden degenerated lutein cell towards centre. Fifty weeks after start of DMB treatment. ×85.

FIG. 15.—A large number of intact corpora lutea. Some of the follicles are undergoing atresia. No effect of the treatment visible. Fourteen weeks after start of DB treatment. (Compare with Fig. 7 and 9 from DMB treated mice in the same age group.) ×30.

1

2

3

5

6

7

8

9

10

11

12

13

14

15

Mody.

including 5 with cystic right ovaries. There was atrophy in one or both ovaries in 2 of the 13 mice treated with MC for 16 weeks. Small fibrous scars together with hyalinisation in the walls of arterioles and vascular dilatation were noticed in these MC-treated ovaries.

### C. *Processes Leading to Senile Atrophy*

The ovaries of mice treated with DB were similar to those of normal virgin mice at comparable ages (Fig. 15), i.e. the changes characteristic of senility with only minor differences took place (Table III). Graafian follicles were somewhat

TABLE III.—*Microscopical Appearances of DB-treated Ovaries*

| | Survival following start of treatment* | 0–15 | 16–33 | 34–51 | >52 |
|---|---|---|---|---|---|
| | Number of mice | 17 | 12 | 24 | 7 |
| Germinal epithelium | Anovular buds | − | + | + | ++ |
| | Dark staining cells | + | + | ++ | ++ |
| Follicles | Primordial | +++ | + | + | − |
| | Graafian | ++ | − | + | − |
| | Atretic follicles | +++ | + | − | − |
| | Atretic remnants | +++ | + | + | + |
| Corporal lutea | New | +++ | + | − | − |
| | Old | ++++ | +++ | + | − |
| | Degenerating (early) | + | + | ++++ | +++ |
| | Degenerated | − | − | + | +++ |
| | Pigment | − | + | ++++ | ++++ |
| | Fibrous scars | − | − | + | + |

* Add 16 weeks for actual age. (Compare with Table I, Mody, 1960.)

more frequent at older ages than in normal ovaries of the same ages. Cystic follicles without haemorrhage, anovular follicles and corpora lutea atretica, i.e. atretic follicles containing lipoids (Fekete, 1946), were occasionally observed while they were rarely observed in normal ovaries. Intact young and old corpora lutea persisted until about 30 weeks after treatment i.e. until 46 weeks of age. Involution commenced within 4 weeks following the beginning of treatment and the content of degenerating (those undergoing early degeneration) and completely degenerated corpora lutea was greater than in normal ovaries, where more lipochrome pigment was present. The old DB-treated ovaries showed fibrous scarring and hyaline degeneration in the walls of small arterioles, these changes being uncommon in normal ovaries. Thus after DB treatment, total loss of follicles and diffuse and thecal luteinisation were absent (Fig. 4). Prominence of the germinal epithelium was noted with age but proliferation and invaginations were less evident than in normal ovaries. Senile atrophy was seen in 20 of the mice treated with DB, the number of pinhead or greatly reduced ovaries being larger than in normal mice.

### *Uterus*

On microscopical examination, the state of the uterus was classed as atrophic, normal or having cystic hyperplasia. In 60 DMB-treated mice, ovarian tumours were present in 12 and cystic hyperplasia occurred in 5 of these (Table IV). By contrast, in 48 mice without ovarian tumours, cystic hyperplasia of a lesser degree occurred in 3 mice. There is thus an association between ovarian tumours and cystic hyperplasia. When the size of the ovarian tumour was considered, it was found that the largest were often not accompanied by cystic hyperplasia. Bali

TABLE IV.—*State of the Uterus and Distribution of Breast Tumours in Ovarian Tumour-bearing and Non-tumour-bearing Mice*

| | | I. DMB | II. BP | III. MC | IV. DB |
|---|---|---|---|---|---|
| Ovarian tumour present | Cystic hyperplasia | 1 | 1 | – | – |
| | Cystic hyperplasia + breast tumour | 4 | – | – | – |
| | Normal uterus + breast tumour | 5 | – | – | – |
| | Normal uterus without breast tumour | 2 | 3 | – | – |
| Ovarian tumour absent | Cystic hyperplasia | 2 | 3 | 2 | 2 |
| | Cystic hyperplasia + breast tumour | 1 | – | 1 | 2 |
| | Normal uterus + breast tumour | 5 | 1 | 5 | 6 |
| | Atrophy of uterus | – | 6 | 3 | 3 |
| | Atrophy of uterus + breast tumour | – | – | 6 | 1 |

and Furth (1949) made a similar observation and suggested that the oestrogenic effects come to a standstill when the tumours reach about 2 cm. in diameter.

### *Vaginal Smears*

In view of the occurrence of spontaneous pseudopregnancy in normal IF virgins kept in fours, a 3-week study of the vaginal smear was made in small groups of treated mice 2 months after the start of treatment. Great individual variation and irregularity in the length of the cycles was noticed among DMB-, MC- and DB-treated mice. Dioestrus was long and oestrus short. By contrast, in BP-treated mice a short 6–7 day cycle occurred, with oestrus of about 2 days, and there was no mucification of the vagina at dioestrus. The cycle in BP-treated mice is thus possibly different from that of normal virgins and similar to that of anosmic mice (Mody, 1960). Further evidence is necessary to substantiate this.

### *Mammary Tumours*

Of 60 DMB-treated mice, ovarian and breast tumours were coincident in 9 out of 12, whereas breast tumours occurred in the absence of ovarian tumours in 6 out of 48 mice (Table IV). In BP-treated mice, none of the 4 ovarian tumours was associated with a breast tumour. Twelve MC-and 9 DB-induced breast tumours occurred in the absence of ovarian tumours. Thus, although there was a high association between breast and ovarian tumours in DMB-treated mice, this association did not hold for the other 3 carcinogens. Howell *et al.* (1954) found that DMB-induced ovarian and breast tumours were dissociated, the distribution being random.

### DISCUSSION

### *Incidence of ovarian tumours*

The occurrence of 12 ovarian tumours in 60 IF mice following treatment with DMB is a lower incidence than that of 53 out of 88 previously reported by Howell

*et al.* (1954). In the present experiments the mice were killed at pre-determined intervals and it is to be expected that the incidence would have been higher had the animals been allowed full survival. Ovarian tumours induced by BP have not been reported previously. No unequivocal tumours were obtained with MC or DB, although pre-tumourous changes followed treatment with MC.

*Sequence of ovarian changes*

The first ovarian effect of DMB, BP or MC is damage to the ovum and this is followed by degeneration of all the existing follicles and failure of new follicles to develop. These effects are rather more rapid with DMB than with the other two chemicals. Abnormalities of luteinisation are followed by the appearance of nodules (Fig. 9 and 10). These are derived from theca-lutein cells and are thought to be the starting point of the tumours of the granulosa cell series. These nodules may be bilateral, although the tumours are always unilateral, and it is therefore suggested that some regress. The final stage in the development of the nodules into tumours was seen after DMB and BP treatment but was not reached after MC treatment in these experiments. From histological examination it is not possible to be certain when the growth of the nodules becomes autonomous. If nodules fail to develop, or regress, the ovaries undergo regressive changes characterised chiefly by proliferation of the cells of the germinal epithelium, which stream inwards to replace the lutein tissue.

By contrast, the changes observed after DB treatment resemble those seen in ageing normal virgins. Total loss of follicles did not occur, abnormal types of luteinisation and nodules were absent and there were no tumours. The specificity of action on the ovary of these chemicals, all of which are carcinogenic to other organs (e.g. the skin), is thus apparent.

The histogenesis of the tumours induced by DMB and BP is similar to that seen in irradiated ovaries (Brambell and Parkes, 1927; Giest, Gaines and Pollack, 1939) and in intrasplenic ovarian grafts (Biskind and Biskind, 1949). The sequence of events after chemicals may include a transitional luteomatous stage but this phase seems to be less persistent than that observed by Lipschutz (1960) and Lipschutz, Rojas, Cerisola and Iglesias (1960) in intrasplenic or fragmented ovaries. From the present experiments it appears that granulosa cell tumours can arise from areas of abnormal luteinisation, without an intervening luteomatous phase, but this is not certain.

*Relation between the occurrence of ovarian and breast tumours*

Following DMB, ovarian and breast tumours frequently occurred in the same mouse but there was no such association when the carcinogen was BP, MC or DB. Marchant (1959) made the interesting observation that ovarian tumours did not develop in normal mouse ovaries grafted into DMB-treated mice, although breast tumours did occur, and that the ovaries of treated mice did develop ovarian tumours when grafted into normal mice.

*Hormonal effects of the ovarian tumours*

Of the 12 mice bearing DMB-induced tumours, there were 5 in which cystic hyperplasia of the uterus was observed (Table IV), and it was also present in the one mouse with a large BP-induced tumour. However, it is an inconstant feature and can only be regarded as a crude index of secretory activity, depending on the ratio of oestrogen to progesterone rather than upon actual quantities. When the BP-induced tumour was grafted into male mice evidence of a feminising effect was seen in lobular development in the breast, suppression of spermatogenesis and lack of secretion in the seminal vesicles.

### SUMMARY

Limited skin applications of four carcinogens (DMB, BP, MC and DB) were made to inbred virgin IF mice, which were subsequently killed at ages ranging from 16 to 70 weeks in order that the sequence of ovarian changes might be studied.

Ovarian tumours of the granulosa-cell series were induced by means of DMB and BP, but not with MC or DB. The induction period was shorter with DMB. Pre-tumorous changes were induced with MC, but DB exerted no effect upon the ovary.

The tumours were unilateral and of the granulosa cell series, the granulosa cell type being predominant. They resembled those occurring spontaneously in some strains of mice, those induced by X-irradiation or in intrasplenic ovarian grafts in castrates and the granulosa cell tumours of the human ovary.

The sequence of histological changes in the ovary after treatment with DMB, BP or MC is death of the ova and degeneration of all the follicles, failure of new follicles to develop, merging of the corpora lutea, proliferation and luteinisation of theca cells and formation of multifocal nodules from these luteinised theca cells in one or both ovaries. The tumours arise unilaterally in one or more nodules, the remainder of which undergo regression. The secondary proliferation and luteinisation of the theca cells following merging of the corpora lutea, with subsequent nodule formation, is regarded as the essential precursor of tumour formation. The ovary contralateral to the tumour-bearing ovary, or both ovaries where no tumour is present, undergoes reduction in size. This is characterised by a streaming into the substance of the ovary of dark staining cells derived from the germinal epithelium and the accumulation of lipochrome pigment in phagocytes.

Following treatment with DB the normal age changes which take place in virgins occur (Mody, 1960). Large cystic follicles, persistent corpora lutea, fibrous scars and hyaline degeneration of blood vessel walls are more frequent than in the normal and some ovaries become greatly reduced in size.

### REFERENCES

Bali, T. and Furth, J.—(1949) *Cancer Res.*, **9**, 449.
Biskind, G. R. and Biskind, M. S.—(1949) *Amer. J. clin. Path.*, **19**, 501.
Brambell, F. W. and Parkes, A. S.—(1927) *Proc. Roy. Soc.* B, **101**, 316.
Fekete, E.—(1946) *Cancer Res.*, **6**, 263.
Giest, S. H., Gaines, J. A. and Pollack, A. D.—(1939) *Amer. J. Obstet. Gynec.*, **38**, 786.
Howell, J. S., Marchant, J. and Orr, J. W.—(1954) *Brit. J. Cancer*, **8**, 635.
Lipschutz, A.—(1960) *Acta. Un. int. Cancr.*, **16**, 149.
*Idem*, Rojas, G., Cerisola, H. and Iglesias, R. (1960) *Ibid.*, **16**, 206.
Marchant, J.—(1957) *Brit. J. Cancer*, **11**, 452.—(1959) *Ibid.*, **13**, 306.
Mody, J.—(1960) Thesis presented for the degree of Ph.D. of Leeds University.